# Supplementary material for: RhopH2 and RhopH3 export enables assembly of the RhopH complex on P. falciparum-infected erythrocyte membranes
Source: Commun Biol. 2022 Apr 7;5:333. doi: 10.1038/s42003-022-03290-3 (PMC8989874; doi:10.1038/s42003-022-03290-3)
Supplement: Supplementary file 2 — Supplementary Material [file 42003_2022_3290_MOESM2_ESM.pdf]

Supplementary information

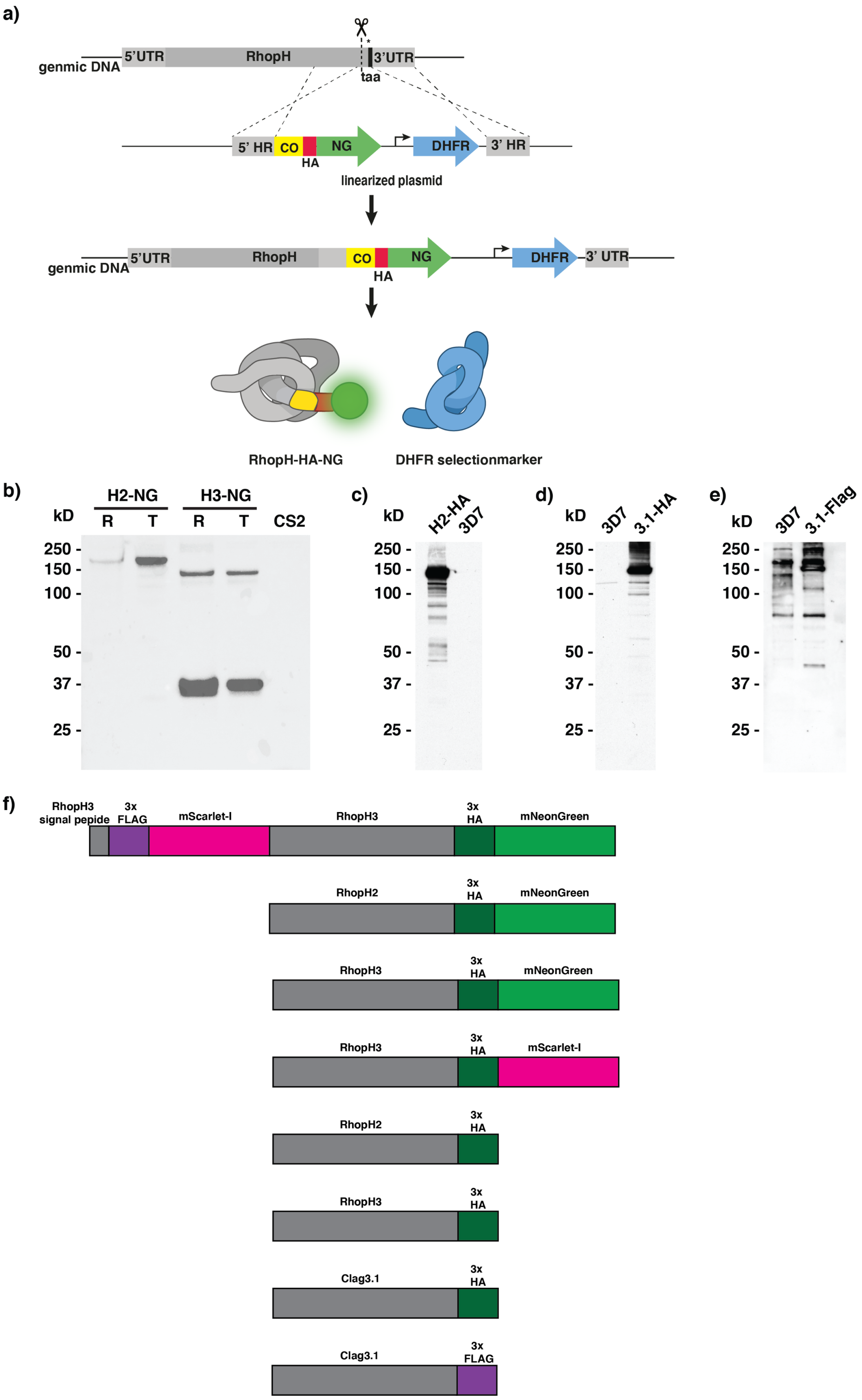

Supplementary Figure 1. CRISPR-Cas9 mediated tagging of RhopH2 and RhopH3

(a) Schematic representation of strategy to tag of RhopH proteins with a triple HA and mNeonGreen. Constructs contain 5' and 3' homology regions (HRs) with codon optimized

region (CO), the triple-HA and mNeonGreen (NG) tag, and hDHFR drug selection marker. Recombination of the homology regions with the homologous sequences in the genomic DNA enables construct integration and the tagging of the endogenous protein. We didn't manage to obtain fluorescently-tagged Clag3.1 but the same approach was utilised to tag it with HA or Flag-tags. (b-e) Western blot confirmation of expression of the RhopH2-mNeonGreen or RhopH3-HA-mNeonGreen (b), RhopH2-HA (c), Clag3.1-HA (d) or Clag3.1-Flag (e) showing expression of the tagged-proteins. Size marker is indicated on the left. 3D7 are the wild-type control parasites. Ribbon representations of protein tagging are shown in (f).

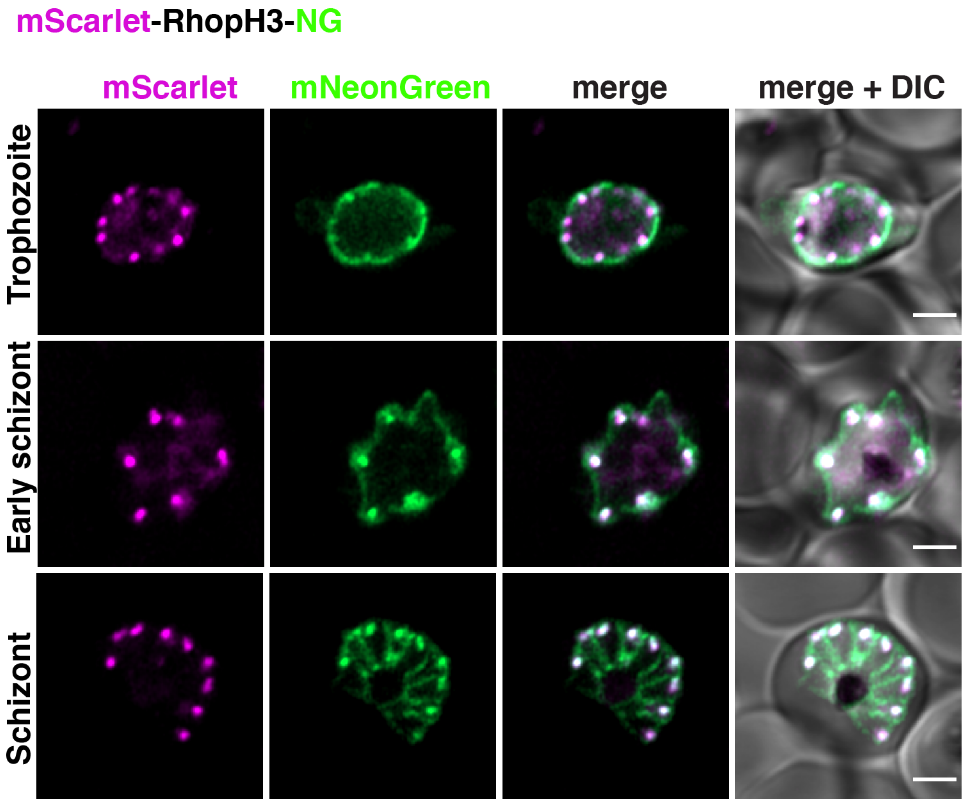

Supplementary Figure 2. Processing of RhopH3 using double-tagged line

Parasites with RhopH3 tagged with mScarlet at the N-terminus and mNeonGreen at the C-terminus were imaged using live super-resolution microscopy revealing the presence of both termini in the rhoptries. This indicates that either full-length RhopH3 or processed RhopH3 with both termini associated are trafficked to rhoptries. On the other hand, membrane associated signal contains no mScarlet and only mNeonGreen suggesting it comes from the cleaved C-terminus. Details in the main body of the manuscript. Scale bar 2 µm.

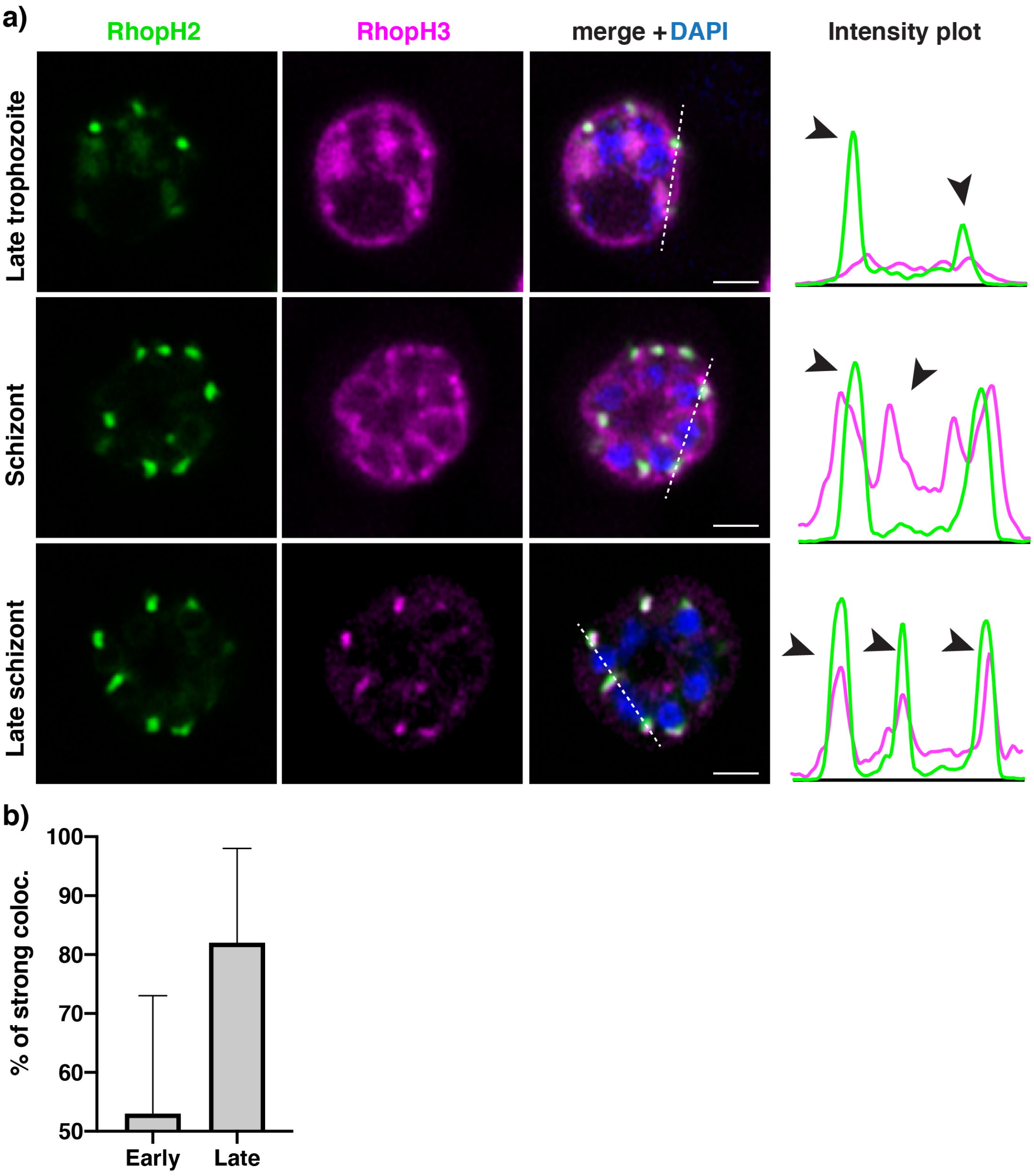

Supplementary Figure 3. RhopH2 and RhopH3 colocalization using live super-resolution microscopy. Parasites expressing both RhopH2-mNeonGreen and RhopH3-mScarlet were imaged live using super-resolution microscopy at trophozoite and early to late schizont stages. Scale bar 2 µm. Intensity plots along the drawn line are displayed on the right side and arrows point to peaks of intensity. The peaks show colocalization of RhopH2 and RhopH3 in the rhoptries. The colocalization is weak at early stages as evident from peaks of one colour (late trophozoite and schizont, arrows). The colocalization of both proteins increases in late schizonts shortly before the egress as evident from the intensity peaks displaying strong signals of both mScarlet and mNeonGreen (a) and the percentage of colocalising loci in early vs late schizonts is quantified in (b).

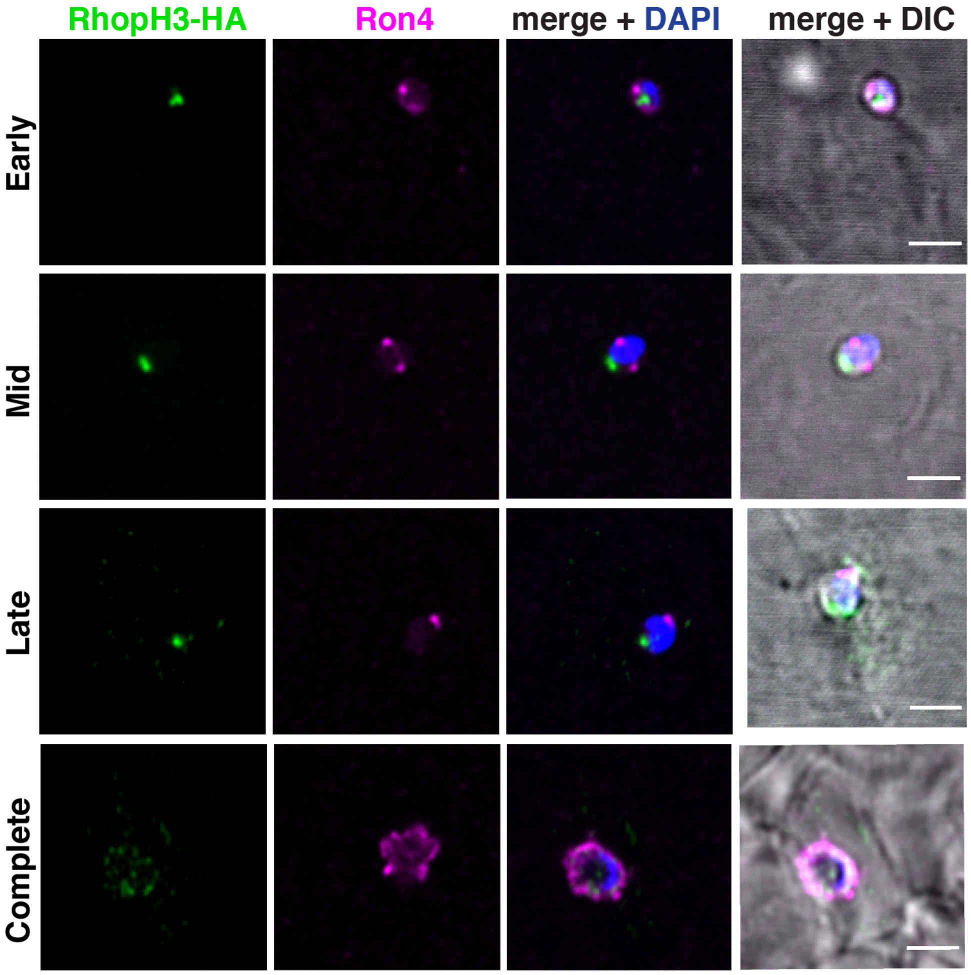

Supplementary Figure 4. The localization of RhopH3-HA-mNeonGeen during invasion

Merozoites of the RhopH3-HA-mNeonGreen were fixed during invasion on red blood cells at 1 min 30 sec or 10 min time points. RON4 (in magenta) was used as a marker of the tight junction to assess the stage of invasion (from early invasion to a complete entry). Scale bar 2 µm. Despite the processing of the C-terminal tag (Supplementary Figure 2), the localization of RhopH3-HA-mNeonGreen is similar to the one in Figure 2 of the main manuscript body, where N-terminally Flag-tagged RhopH2 was used. These data suggest that upon successful invasion, RhopH3 is localised in the newly-formed parasitophorous vacuole.

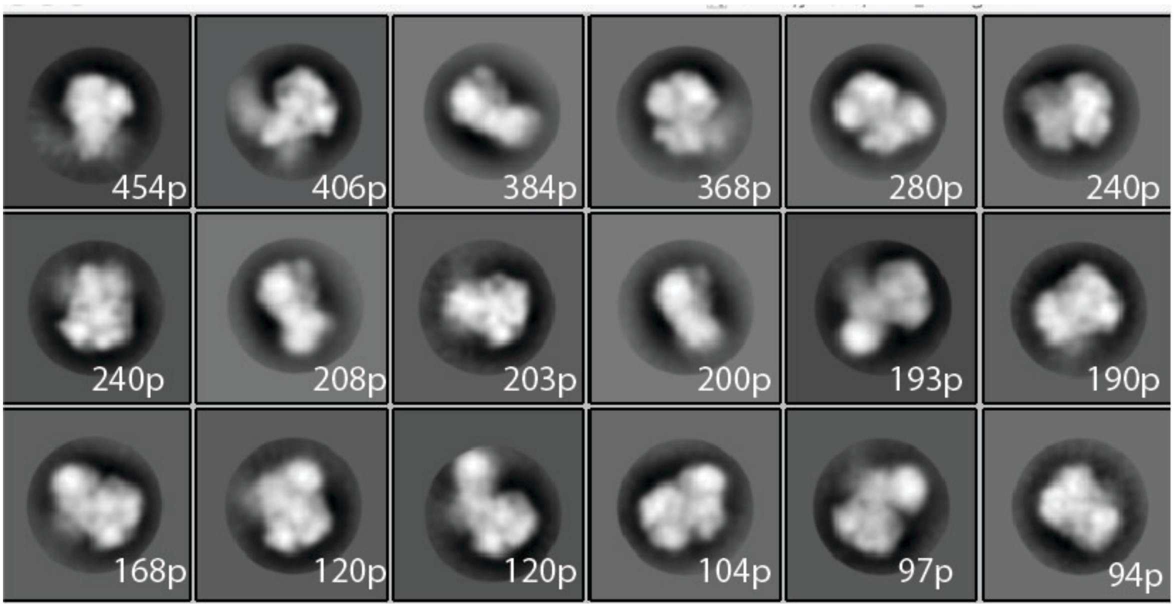

Supplementary Figure 5. Negative stain 2D classes of the RhopH complex

The figure show obtained 2D class averages of the RhopH complex used in the membrane incorporation studies (Figure 5 b and c) and is similar to the recently published structures (Ho *et al*, 2021; Schureck *et al*, 2021). The number of particles that contributed to each class is displayed on the bottom right of each 2D class.

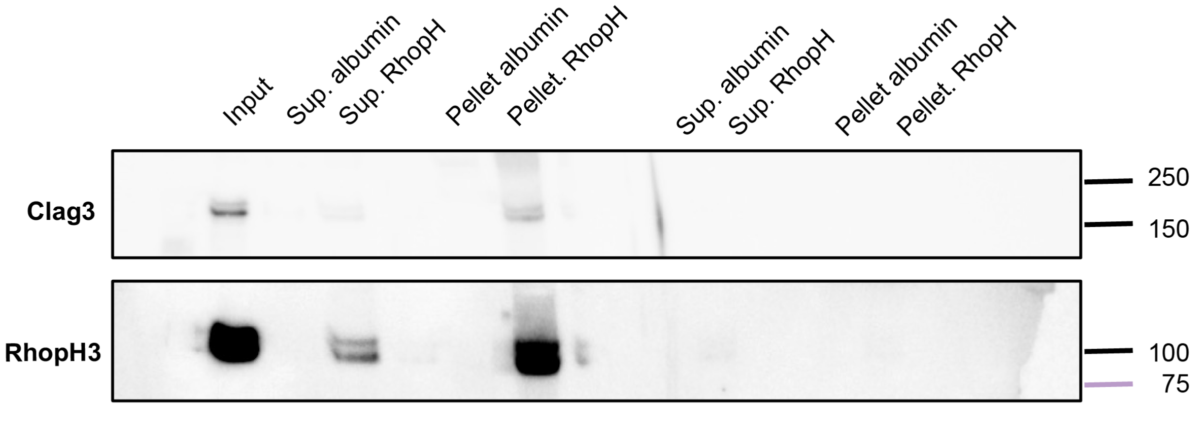

Supplementary Figure 6. RhopH complex association with RBC ghosts and liposomes. Purified RhopH complex associates with red blood cell ghosts but not with synthetic liposomes as evident from the lack of RhopH3 and Clag3 in the liposome pellet. The lack of detectable signal in the liposome supernatant stems from the large volume necessary to wash and spin the liposomes in the ultracentrifuge which led to the dilution of the protein during the wash-out.

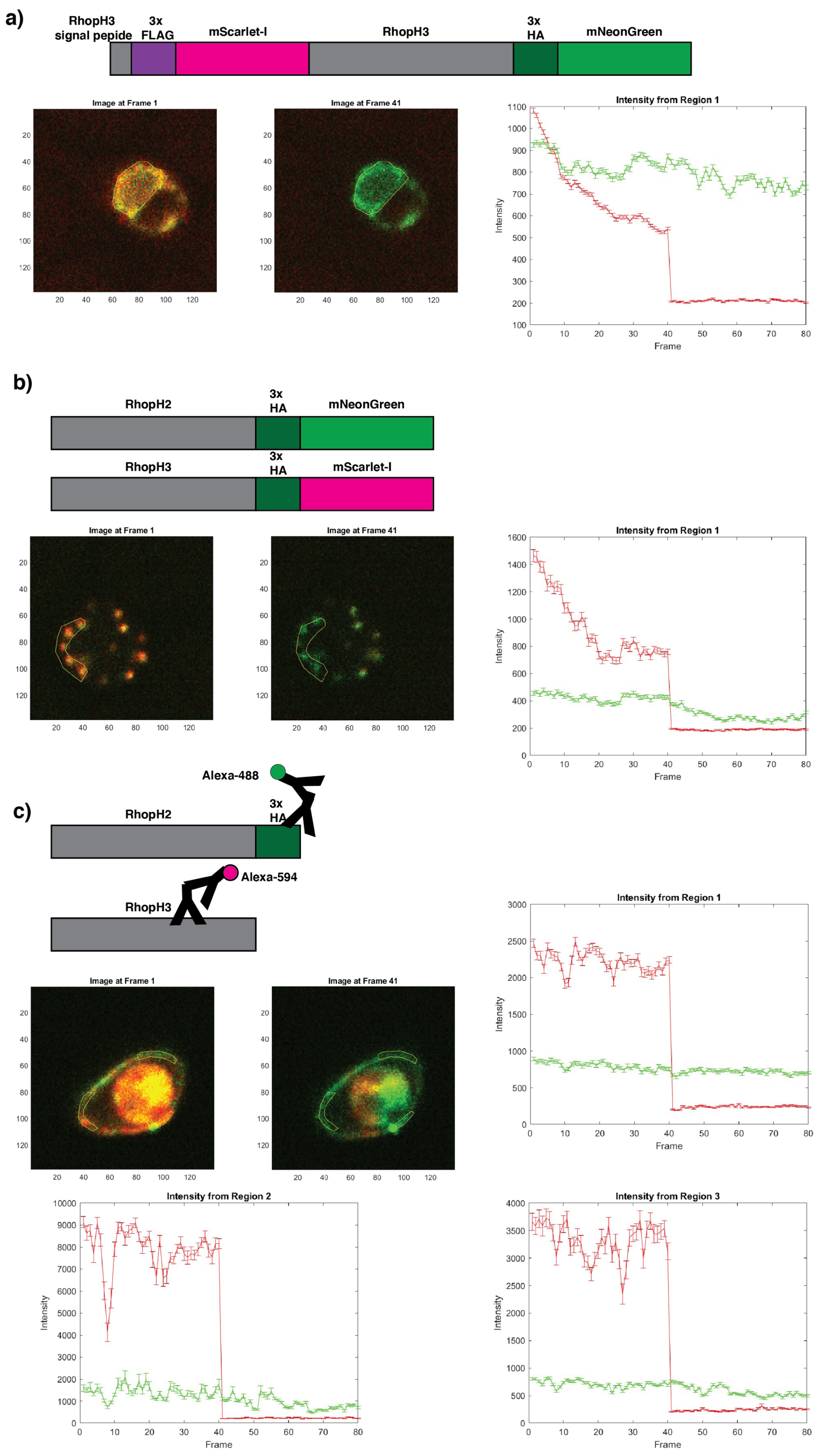

Supplementary Figure 7. FRET attempt in double-tagged parasite lines.

Images before and after photobleaching of the acceptor and measurements of the fluorescent intensity in the marked regions. a) parasite line where RhopH3 with two fluorophores: mScarlet at the N terminus and mNeonGreen at the C terminus; b) RhopH2 was tagged with mNeonGreen and RhopH3 was tagged with mScarlet, both tags are at the C termini; c) RhopH2-HA and RhopH3 were detected using immunofluorescence.

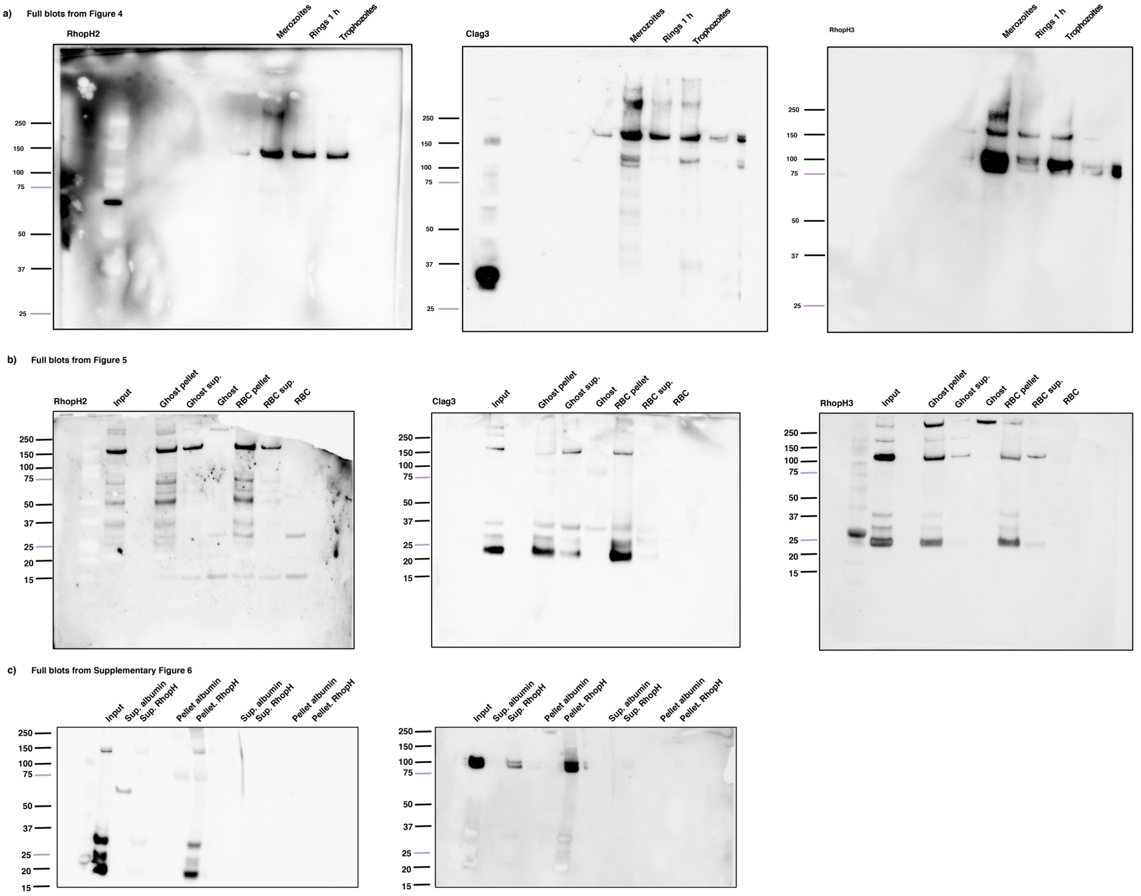

Supplementary Figure 8. RhopH full blots for Figures 4, 5 and Supplementary Figure 6. Protein ladder shows molecular weights in kDa.

Supplementary Table 1. List of proteins detected by Mass Spectrometry following RhopH-HA pulldown from various developmental stages: free merozoites, rings and trophozoites. Wild type 3D7 parasites were used as a control. The table shows the log2 fold change compared to the control sample and the values have been colour-coded with the highest values in blue and the lowest in red. Statistical significance (p-value) is shown on the right and results defined as real changes have been highlighted on the right-hand side as TRUE (light green) and FALSE (red) for not-statistically significant differences. Each sample is an average of 3 biological replicates.

Supplementary Table 2. List of proteins detected by Mass Spectrometry in the purified RhopH complex following size-exclusion chromatography (Figure 5). This complex was used in the membrane incorporation studies (Figure 5b and 5c).

Supplementary Movie 1. Super-resolution live imaging RhopH2-mNeonGreen expressing parasite with SiR-DNA-stained nuclei (blue). mNeonGreen signal accumulation in cytoplasm of a late trophozoite and in the forming rhoptries. Scale bar 2 µm, time points every 15 min.

Supplementary Movie 2. Super-resolution live imaging RhopH3-mNeonGreen expressing parasite with SiR-DNA-stained nuclei (blue). mNeonGreen signal accumulation in cytoplasm of a late trophozoite and in the forming rhoptries. A membrane-associated signal is present until close to the egress. Scale bar 2 µm, time points every 20 min.

Supplementary Movie 3. Subcellular localisation of RhopH2 during parasite invasion. Live RhopH2-mNeonGreen merozoites invading human erythrocytes imaged using lattice-light sheet microscopy. mNeonGreen signal visible as a bright spot on the merozoite apical end corresponding to RhopH2. The signal became diffused upon successful invasion as the parasitophorous vacuole formed. Scale bar 2 µm. Parasites stained with mitotracker deep red (cyan) and red blood cells stained with Di-4-ANEPPDHQ (magenta).

Supplementary Movie 4. Subcellular localisation of RhopH3 during parasite invasion. Live RhopH3-mNeonGreen merozoites invading human erythrocytes imaged using lattice-light sheet microscopy. mNeonGreen signal visible as a bright spot on the merozoite apical end corresponding to RhopH3. The signal became diffused upon successful invasion as the parasitophorous vacuole formed. Scale bar 2 µm. Parasites stained with mitotracker deep red (cyan) and red blood cells stained with Di-4-ANEPPDHQ (magenta).

Supplementary methods

Liposome preparation

Liposomes were prepared with lipid mixture approximating the composition of inner leaflet of human erythrocyte membrane as reported by Virtanen et al, 1998. Briefly, 14% 1-palmitoyl-2-oleoyl-sn-glycero-3-phosphocholine (POPC), 9.1% Sphingomyelin, 43.9% 1-Palmitoyl-2-Oleoyl-sn-Glycero-3-Phosphoethanolamine (POPE), 29.6% 1,2-dioleoyl-sn-glycero-3-phospho-L-serine (DOPS), 1.2% L-α-phosphatidylinositol (PI), and 2.2% 3-sn-Phosphatidic

acid (PA) chloroform solutions were mixed and dried under N2 gas. Lipid mixture was resuspended in 10 mM HEPES pH 7.5, 135 mM KCl, 1 mM MgCl2. Resuspended lipids were frozen and thawed several times and then extruded an odd number of times (more than 20) through a membrane with a pore size of 100 nm. The resulting liposomes were stored at 4 °C. POPE was purchased from Anatrace, PA from sigma and the rest of the lipids from Avanti Polar lipids Inc.

FRET

Samples were imaged on a Zeiss 980 confocal microscope with a 63x 1.4 NA oil immersion objective. Both the donor (mNeonGreen or Alexa-488) and acceptor (mScarlet or Alexa-594) channels were imaged for either 20 or 40 frames before the acceptor was photobleached. Both channels were imaged for 20 or 40 frames after acceptor photobleaching. The mean intensity of the photobleached region in both channels were calculated for the entire time series.
