# Supplementary material for: RhopH2 and RhopH3 export enables assembly of the RhopH complex on P. falciparum-infected erythrocyte membranes
Source: Commun Biol. 2022 Apr 7;5:333. doi: 10.1038/s42003-022-03290-3 (PMC8989874; doi:10.1038/s42003-022-03290-3)
Supplement: Supplementary file 3 — Description of Additional Supplementary Files [file 42003_2022_3290_MOESM3_ESM.pdf]

Description of Additional Supplementary Files

File name: Supplementary Data 1.
Description: List of proteins detected by Mass Spectrometry following RhopHHA pulldown from various developmental stages: free merozoites, rings and trophozoites.

Wild type 3D7 parasites were used as a control. The table shows the log2 fold change compared to the control sample and the values have been colour-coded with the highest values in blue and the lowest in red. Statistical significance (p-value) is shown on the right and results definied as real changes have been highlighted on the right-hand side as TRUE (light green) and FALSE (red) for not-statistically significant differences. Each sample is an average of 3 biological replicates.

File name: Supplementary Data 2.
Description: List of proteins detected by Mass Spectrometry in the purified RhopH complex following size-exclusion chromatography (Figure 5). This complex was used in the membrane incorporation studies (Figure 5b and 5c).

File name: Supplementary Movie 1
Description: Super-resolution live imaging RhopH2-mNeonGreen expressing parasite with SiR-DNA-stained nuclei (blue). mNeonGreen signal accumulation in cytoplasm of a late trophozoite and in the forming rhoptries. Scale bar 2 µm, time points every 15 min.

File name: Supplementary Movie 2
Description: Super-resolution live imaging RhopH3-mNeonGreen expressing parasite with SiR-DNA-stained nuclei (blue). mNeonGreen signal accumulation in cytoplasm of a late trophozoite and in the forming rhoptries. A membrane-associated signal is present until close to the egress. Scale bar 2 µm, time points every 20 min.

File name: Supplementary Movie 3
Description: Subcellular localisation of RhopH2 during parasite invasion. Live RhopH2-mNeonGreen merozoites invading human erythrocytes imaged using lattice-light sheet microscopy. mNeonGreen signal visible as a bright spot on the merozoite apical end corresponding to RhopH2. The signal became diffused upon successful invasion as the parasitophorous vacuole formed. Scale bar 2 µm. Parasites stained with mitotracker deep red (cyan) and red blood cells stained with Di-4-ANEPPDHQ (magenta).

File name: Supplementary Movie 4
Description: Subcellular localisation of RhopH3 during parasite invasion. Live

RhopH3-mNeonGreen merozoites invading human erythrocytes imaged using lattice-light sheet microscopy. mNeonGreen signal visible as a bright spot on the merozoite apical end corresponding to RhopH3. The signal became diffused upon successful invasion as the parasitophorous vacuole formed. Scale bar 2 µm. Parasites stained with mitotracker deep red (cyan) and red blood cells stained with Di-4-ANEPPDHQ (magenta)
