# Supplementary material for: RhopH2 and RhopH3 export enables assembly of the RhopH complex on P. falciparum-infected erythrocyte membranes
Source: Commun Biol. 2022 Apr 7;5:333. doi: 10.1038/s42003-022-03290-3 (PMC8989874; doi:10.1038/s42003-022-03290-3)
Supplement: Supplementary file 10 — Reporting Summary [file 42003_2022_3290_MOESM10_ESM.pdf]

nature portfolio

Corresponding author(s): Alan F. Cowman

Last updated by author(s): Dec 29, 2021

Reporting Summary

Nature Portfolio wishes to improve the reproducibility of the work that we publish. This form provides structure for consistency and transparency in reporting. For further information on Nature Portfolio policies, see our Editorial Policies and the Editorial Policy Checklist.

Statistics

For all statistical analyses, confirm that the following items are present in the figure legend, table legend, main text, or Methods section.

n/a	Confirmed	
☐	☒	The exact sample size (n) for each experimental group/condition, given as a discrete number and unit of measurement
☐	☒	A statement on whether measurements were taken from distinct samples or whether the same sample was measured repeatedly
☐	☒	The statistical test(s) used AND whether they are one- or two-sided<br>*Only common tests should be described solely by name; describe more complex techniques in the Methods section.*
☒	☐	A description of all covariates tested
☒	☐	A description of any assumptions or corrections, such as tests of normality and adjustment for multiple comparisons
☐	☒	A full description of the statistical parameters including central tendency (e.g. means) or other basic estimates (e.g. regression coefficient) AND variation (e.g. standard deviation) or associated estimates of uncertainty (e.g. confidence intervals)
☒	☐	For null hypothesis testing, the test statistic (e.g. F, t, r) with confidence intervals, effect sizes, degrees of freedom and P value noted<br>*Give P values as exact values whenever suitable.*
☒	☐	For Bayesian analysis, information on the choice of priors and Markov chain Monte Carlo settings
☒	☐	For hierarchical and complex designs, identification of the appropriate level for tests and full reporting of outcomes
☒	☐	Estimates of effect sizes (e.g. Cohen's d, Pearson's r), indicating how they were calculated

Our web collection on statistics for biologists contains articles on many of the points above.

Software and code

Policy information about availability of computer code

Data collection	For Lattice Light Sheet imaging data was deskewed and deconvolved using LLSpy, a Python interface.<br>Deconvolution was performed using a Richardson-Lucy algorithm using the PSFs generated for each excitation wavelength.
Data analysis	ImageJ version 2.3.0/1.53n

For manuscripts utilizing custom algorithms or software that are central to the research but not yet described in published literature, software must be made available to editors and reviewers. We strongly encourage code deposition in a community repository (e.g. GitHub). See the Nature Portfolio guidelines for submitting code & software for further information.

Data

Policy information about availability of data

All manuscripts must include a data availability statement. This statement should provide the following information, where applicable:

- Accession codes, unique identifiers, or web links for publicly available datasets
- A description of any restrictions on data availability
- For clinical datasets or third party data, please ensure that the statement adheres to our policy

The datasets generated during and/or analysed during the current study are available from the corresponding author on reasonable request.

Field-specific reporting

Please select the one below that is the best fit for your research. If you are not sure, read the appropriate sections before making your selection.

☒ Life sciences ☐ Behavioural & social sciences ☐ Ecological, evolutionary & environmental sciences

For a reference copy of the document with all sections, see nature.com/documents/nr-reporting-summary-flat.pdf

Life sciences study design

All studies must disclose on these points even when the disclosure is negative.

Sample size	N/A
Data exclusions	No data was excluded.
Replication	Where appropriate all experiments were repeated at least three times independently.
Randomization	N?A
Blinding	N/A

Reporting for specific materials, systems and methods

We require information from authors about some types of materials, experimental systems and methods used in many studies. Here, indicate whether each material, system or method listed is relevant to your study. If you are not sure if a list item applies to your research, read the appropriate section before selecting a response.

Materials & experimental systems

n/a	Involved in the study	
☐	☒	Antibodies
☐	☒	Eukaryotic cell lines
☒	☐	Palaeontology and archaeology
☒	☐	Animals and other organisms
☒	☐	Human research participants
☒	☐	Clinical data
☒	☐	Dual use research of concern

Methods

n/a	Involved in the study	
☒	☐	ChIP-seq
☒	☐	Flow cytometry
☒	☐	MRI-based neuroimaging

Antibodies

Antibodies used	We used the following antibodies (also specified in the Methods section): Monoclonal antibodies: rat anti-HA (Roche 3F10, Cat.: 11867423001, Lot: 47877600), mouse anti-FLAG (Sigma M2, Cat.: F1804, Lot: SZCD3524), or mouse monoclonal antibodies produced in-house: anti-HA (12CA5), anti-FLAG 9H1. Polyclonal antibodies against RhopH2, RhopH3 and Clag3 were generated in rabbits by GenScript against the following antigens: RhopH3 T731-Y829, RhopH2 L20-S1378 and Clag3 K1277-H1417 and verified using indirect ELISA by the manufacturer. Rabbit anti-RON4 serum made in house and published by us previously: Richard D, et al. Interaction between Plasmodium falciparum apical membrane antigen 1 and the rhoptry neck protein complex defines a key step in the erythrocyte invasion process of malaria parasites. J Biol Chem 285, 14815-14822 (2010). The following secondary Alexa 488/594 fluorophores from Life Technologies were used: chicken anti-mouse 594 (Cat.: A21201, Lot: 42099A), donkey anti-rat 488 (Cat.: A21208, Lot: 2310102), chicken anti-rabbit 594 (Cat.: A21442, Lot: 2110863).The following secondary HRP-conjugated antibodies were used: goat a-rat (Southern Biotech, Cat.: 3030-05, Lot: G2512-M748B), goat [illegible]-mouse (Merck Millipore, Cat: AP124P), goat a-rabbit (Merck Millipore, Cat: AP187P).
Validation	Validation by the manufacturer as per manufacturer's website. Polyclonal rabbit antibodies produced by GenScript were validated by the manufacturer using indirect ELISA.

Eukaryotic cell lines

Policy information about cell lines

Cell line source(s)	3D7 and CS2 P. falciparum lines
Authentication	These lines are periodically sequenced for other projects but this serves as an authentication that they are the expected versions of the P. falciparum lines.

Mycoplasma contamination	P. falciparum lines are tested periodically for Mycoplasma infection and have are negative.
Commonly misidentified lines (See ICLAC register)	N/A
